# Biological control of tomato bacterial wilt and apple fire blight through the induced resistance of azomycin derived from *Streptomyces* sp. JCK-8368

**DOI:** 10.3389/fpls.2025.1654826

**Published:** 2025-09-26

**Authors:** Loan Thi Thanh Nguyen, Ae Ran Park, Hye Won Im, Ve Van Le, Hang T. T. Nguyen, Quang Le Dang, Tran Thi Nhu Hoa, Yu Jeong Yeo, Ha Hang Le, Van Thi Nguyen, Inmin Hwang, Jin-Cheol Kim

**Affiliations:** ^1^ Plant Healthcare Research Institute, JAN153 Biotech Incorporated, Jeongeup, Republic of Korea; ^2^ Department of Agricultural Chemistry, College of Agriculture and Life Sciences, Institute of Environmentally Friendly Agriculture, Chonnam National University, Gwangju, Republic of Korea; ^3^ Faculty of Biotechnology, College of Applied Life Sciences, Jeju National University, Jeju, Republic of Korea; ^4^ Faculty of Applied Sciences, Ton Duc Thang University, Ho Chi Minh City, Vietnam; ^5^ Institute of Materials Sciences, Vietnam Academy of Science and Technology, Hanoi, Vietnam; ^6^ Graduate University of Science and Technology, Vietnam Academy of Science and Technology, Hanoi, Vietnam; ^7^ Plant Clinic, Vietnam National University of Agriculture, Hanoi, Vietnam; ^8^ Hygienic Safety and Analysis Center, World Institute of Kimchi, Gwangju, Republic of Korea

**Keywords:** tomato bacterial wilt, apple fire blight, *Streptomyces* sp., biocontrol agent, azomycin, plant defense resistance

## Abstract

Tomato bacterial wilt and apple fire blight, caused by *Ralstonia solanacearum* and *Erwinia amylovora*, respectively, are highly destructive diseases that threaten global agriculture productivity. Increasing resistance of these pathogens to conventional antibiotics and copper-based pesticides highlights the urgent need for sustainable, eco-friendly biocontrol alternatives. This study aimed to evaluate the biocontrol potential of the azomycin-producing *Streptomyces* sp. JCK-8368 (hereafter JCK-8368) against tomato bacterial wilt and apple fire blight, and to investigate its possible resistance-inducing mechanism. The culture filtrate (CF) of JCK-8368, containing azomycin, was applied to the plant at 1,000-fold (100 ng/mL), 500-fold (200 ng/mL), and 250-fold (400 ng/mL) dilutions via foliar spraying or soil drenching. Purified azomycin was tested at concentrations from 1 ng/mL to 1000 ng/mL. Disease severity and control efficacy were assessed, and expression of defense-related genes (*PR1*, *PR2*, *PR3*, and *PR5*) was also analyzed. Foliar spraying and soil drenching with JCK-8368 CF significantly reduced tomato bacterial wilt severity, with control efficacies of 52.22% (1000-fold), 11.11% (500-fold), and 35.55% (250-fold) in foliar application, 90.00%, 77.78%, and 52.22% in soil drenching, respectively. The reversed dose-response pattern in soil drenching indicated higher efficacy at lower concentrations. In apple fire blight control, soil drenching with CF at a 1,000-fold dilution achieved foliar spraying (78.38%) efficacy, exceeding soil drenching (50.88%). In particular, purified azomycin most effectively reduced tomato bacterial wilt at 100 ng/mL (57.14% efficacy) and showed a clear dose-dependent effect from 1 to 100 ng/mL. The plants treated with JCK-8368 CF and azomycin upregulated defense-related genes such as *PR1*, *PR2*, *PR3*, and *PR5*, suggesting systemically acquired resistance and pathogenesis-related defense pathways. This is the first report demonstrating the application of azomycin against plant bacterial diseases, showing that low concentrations of JCK-8368 and purified azomycin can effectively control tomato bacterial wilt and apple fire blight through induced resistance. Azomycin-producing *Streptomyces* sp. JCK-8368 offers a promising, sustainable alternative to chemical pesticides, warranting further field validation and formulation development for agricultural use.

## 1 Introduction

Tomato bacterial wilt and apple fire blight, caused by *Ralstonia solanacearum* (Peeters et al., 2013) and *Erwinia amylovora* (Paulin, 2000), respectively, rank among the most destructive plant diseases worldwide, causing substantial economic losses and severe yield reduction (Mansfield et al., 2012). *R. solanacearum* infects over 450 plant species, including tomatoes, potatoes, peppers, and eggplants, causing yield losses of 30–90% in severely affected regions (Elphinstone, 2005). In Korea, bacterial wilt remains a major threat to agriculture, especially on high-value solanaceous crops, causing substantial yield and revenue losses (Cho et al., 2018). Similarly, *E. amylovora*, the causal agent of apple fire blight, causes catastrophic losses in apple and pear production countries, with individual outbreaks leading to losses exceeding millions of dollars due to tree removal, orchard destruction, and trade restrictions (Bonn and Zwet, 2000). Since its first detection in Korea in 2015, apple fire blight has rapidly expanded, infecting 744 orchards across major apple-growing regions by 2020, causing considerable economic damage and loss of valuable apple cultivars (Lee et al., 2022; Park et al., 2017). The rapid spread of *R. solanacearum* and *E. amylovora* in host plants necessitates research into effective disease management strategies.

Various strategies have been explored to manage these diseases, including chemical treatments, cultural practices, the use of resistant cultivars, genetic modifications, and biological methods (Adhikari et al., 2020). Chemical pesticides traditionally control these diseases, but overuse results in unintended consequences such as soil pollution, toxic residues in food products, a decline in beneficial microorganisms, and the emergence of antibiotic-resistant pathogens (Özkara et al., 2016). These challenges shift focus toward biological control, an environmentally friendly and sustainable alternative for long-term disease suppression (Bonaterra et al., 2022).

Plant-microbe interactions play a critical role in disease management by directly or indirectly suppressing pathogens through complex and dynamic relationships (Ab Rahman et al., 2018; Kumar et al., 2017). Beneficial microbes directly antagonize pathogens by producing bioactive compounds, such as antibiotics, siderophores, and lytic enzymes that inhibit pathogen growth or induce cell death (Dimkić et al., 2022). Indirect antagonism enhances plant defense by activating the innate immune system in the plant (Rojo et al., 2003). Among these beneficial microbes, *Streptomyces* species attract significant attention for producing diverse secondary metabolites with potent antibacterial and antifungal activities (Alam et al., 2022; Oluwaseyi and Babalola, 2019). Moreover, they promote plant growth by colonizing plant roots, combating pathogens, and degrading phytotoxins (Oluwaseyi and Babalola, 2019). Although *Streptomyces* species show promise in managing phytopathogens (Oluwaseyi and Babalola, 2019), for example, *S. griseoviridis* and *S. lydicus* (commercialized as Mycostop and Actinovate, respectively) are used to control fungal pathogens (Bubici, 2018), their potential for controlling tomato bacterial wilt and apple fire blight remains uninvestigated.

Bioactive compounds derived from *Streptomyces* species hold potential for antibiotic and antiparasitic drug production. William Campbell and Satoshi Ōmura were awarded the 2015 Nobel Prize in Physiology or Medicine for discovering and applying the avermectins—antiparasitic drugs isolated from *S. avermitilis* (Tatsuta, 2016). *Streptomyces* species also produce approximately 80% of actinomycete-derived microbial antibiotics, including well-known antibiotics streptomycin, chloramphenicol, tetracycline, actinomycin D, and daptomycin (Alam et al., 2022). Among *Streptomyces* antibiotics, 2-nitroimidazole—a macrolide first isolated from *S. eurocidicus*—is also known as azomycin (Maeda, 1953). This redox-activate antibiotic exhibits broad-spectrum activity against various human pathogenic bacteria and protozoa, including *Trichomonas vaginalis* (Torreele et al., 2010). Therefore, this antibiotic is pharmaceutically used to treat various infections, including amoebiasis and bacterial vaginosis (Müller, 1999). However, its potential utilization in agriculture, particularly—for managing tomato bacterial wilt and apple fire blight—remains unexplored. The compound’s redox-active nature suggests that it may generate reactive oxygen species (ROS), which are key signaling molecules in plant defense pathways (Klessig et al., 2000). ROS not only contribute to the direct suppression of pathogens but also plays pivotal roles in activating systemic resistance through salicylic acid (SA) and jasmonic acid (JA) signaling networks (Gonzalez-Bosch, 2018; Nawrocka et al., 2019). This dual mode of action provides a compelling rationale for investigating azomycin’s potential in plant disease management, both as a direct antimicrobial agent and as a priming signal to enhance host immunity.

Plants possess a complex immune system to defend against pathogens and diseases (Kaur et al., 2022). SA and JA signaling pathways are crucial regulators of plant immunity, activating systemic acquired resistance (SAR) and induced systemic resistance (ISR) (Betsuyaku et al., 2018). These responses help “prime” the plant, placing it in a heightened state of alert for quicker and stronger defense activation when challenged by a pathogen (Torreele et al., 2010). Activation of the SAR and ISR pathways typically upregulates pathogenesis-related (*PR*) genes such as *PR1*, *PR2*, *PR3*, and *PR5*, which contribute to antimicrobial compound production, cell wall strengthening, and inhibition of pathogen spread (Jain and Khurana, 2018; Takahashi et al., 2004). Activating plant defense responses offers an effective strategy for pest control in conventional agriculture (Rodriguez-Saona et al., 2022). Although microorganisms have been employed in previous studies to control *R. solanacearum* and *E. amylovora* (Aktepe and Aysan, 2023; Elsayed et al., 2020; Mikiciński et al., 2020), few reports of the activation of a *PR* gene in tomato or apple seedlings during their intricate interplay with *Streptomyces*, particularly highlighting the role of *Streptomyces* secondary metabolites.

During screening of bacterial strains with antagonistic activity and their ability to induce plant resistance, *Streptomyces* sp. JCK-8368 strain (hereafter referred to as JCK-8368) demonstrates remarkable effectiveness, promoting the initiation of this study. Therefore, this study aims to (i) determine the taxonomic identity of JCK-8368, (ii) identify its active metabolite, (iii) evaluate the disease control efficacy of the metabolites against apple fire blight and tomato bacterial wilt, and (iv) investigate the mechanisms by which JCK-8368 and its metabolite control these diseases. To the best of our knowledge, this study is the first to report the potential of azomycin to control bacterial wilt in tomatoes and fire blight in apples.

## 2 Materials and methods

### 2.1 Isolation, culture conditions, and identification of JCK-8368

JCK-8368 was isolated from the root of pepper (*Capsicum* sp.) collected in Daejeon, Korea, as a part of a screening project described by Nguyen et al. (2024). A total of 418 isolates were obtained and screened for antimicrobial activity against *Ralstonia solanacearum* and *Erwinia amylovora* using a serial broth dilution *in vitro* assay, and for induced resistance potential using a GUS reporter assay in *Arabidopsis thaliana*. JCK-8368 was selected for further study based on its minimum inhibition concentrations (MICs) of culture filtrate ≤10% against both pathogens and positive GUS activity. The strain was cultured following the protocol described by Nguyen et al. (2024). JCK-8368 was cultivated on tryptic soy agar (TSA, Difco, Detroit, USA) and incubated at 28°C for 7 days. Morphological properties were examined using a scanning electron microscope (Quanta™ 250 FEG; FEI Company, Oregon, USA). Biochemical and physiological characteristics of JCK-8368 were determined according to Gang et al. (2019).

The taxonomic position of strain JCK-8368 was identified through 16S ribosomal ribonucleic acid (rRNA) sequencing. The 16S rRNA gene was amplified and sequenced using the universal primer set 27F/1492R (Weisburg et al., 1991). The resulting gene sequence was compared to corresponding sequences of cultured species using the EzTaxon server (http://eztaxon-e.ezbiocloud.net) (**Supplementary Table S1**). Sequences of strain JCK-8368 and its closely related species were aligned using ClustalW (Thompson et al., 1994). A phylogenetic tree was then constructed in MEGA X using the maximum-likelihood (ML) method with 1,000 bootstrap replications (Kumar et al., 2018). The optimal substitution model for the ML analysis was selected based on the lowest Bayesian information criterion score from the Model Test in MEGA. The ML tree was reconstructed using the HKY (Hasegawa KishinoYano) (Hasegawa et al., 1985) with a gamma distribution rate (+G) and invariant sites (+I). The evolutionary distances were computed using the Kimura 2-parameter method (Kimura, 1983). As *Actinomadura* and *Streptomyces* belong to the same phylum (Actinomycetota), share a close evolutionary relationship, yet possess sufficiently distinct characteristics, *Actinomadura madurae* ATCC 19425^T^ was selected as the outgroup.

### 2.2 Isolation and structural characterization of antibacterial secondary metabolite

The antibacterial secondary metabolite of JCK-8368 was sequentially extracted with ethyl acetate (EtOAc) and butanol. The isolation was performed under the antimicrobial bioassay guidance using the plant pathogenic bacterial strain *E. amylovora* TS3128 (**Supplementary Figure S1**). The purity of the compound was evaluated using high-performance liquid chromatography (HPLC) (Waters Alliance e2695 system, Milford, MA, USA) equipped with an Atlantis T3 C18 column (4.6 × 250 mm; Waters, Milford, MA, USA). The mobile phase consisted of a gradient system of 0.1% trifluoroacetic acid (TFA) in water and 0.1% TFA in acetonitrile. The gradient profile, with a flow rate of 1 mL/min, was as follows: 0min (20% acetonitrile), 25min (100% acetonitrile), and 30min (100% acetonitrile).

The chemical structures of the antibacterial metabolites were elucidated using ultra-high-performance liquid chromatography-quadrupole-Orbitrap mass spectrometry (UHPLC-Q-Orbitrap MS), gas chromatography-mass spectrometry (GC-MS), and nuclear magnetic resonance (NMR) spectroscopy (Kim et al., 2024; Le et al., 2022; Nguyen et al., 2024). UHPLC-Q-Orbitrap MS analysis was performed following the protocol described by Kim et al., 2024 with the following modifications: The column was maintained at 40°C and eluted with a multilinear gradient using 0.1% (v/v) formic acid in water (mobile phase A) and acetonitrile with 0.1% formic acid (v/v) (mobile phase B) at a flow rate of 0.2 mL/min. The gradient conditions were established as follows: an initial 2-min hold at 5% mobile phase B, a linear increase in organic composition to 10% mobile phase B over 3min, and a further increase to 15% mobile phase B in 2min, eventually reaching 30% mobile phase B after 3min, and culminating at 100% mobile phase B by 20min. The composition was held at 100% mobile phase B for 2min before returning to the initial condition at 23min. GC-MS analysis of the isolated compounds was performed using a Shimadzu GCMS-QP2010 gas chromatograph (70 eV; Shimadzu Co., Kyoto, Japan) equipped with a DB-5MS capillary column (30m × 0.25mm, 0.25 µm film thickness; Agilent Technologies, Inc., Santa Clara, CA, USA). Helium served as the carrier gas at a flow rate of 1.22 mL/min. The GC analysis temperature program began at 120°C for 1min, then increased to 300°C at a rate of 15°C/min and held for 27min. The mass spectrometer operated in positive electron ionization mode at 70 eV, with a source temperature of 260°C and a scan range of 50–600 *m/z*. The mass spectra of the compounds were compared with available data in the WILEY8 Library for identification (Le et al., 2022). For NMR analysis, ^1^H spectra were obtained in DMSO-*d6* using a Bruker Avance III HD 500 MHz instrument (Bruker Biospin GmbH, Rheinstetten, Germany) (Nguyen et al., 2024).

### 2.3 *In vitro* evaluation of antimicrobial activity

Twelve phytopathogens, representing a broad spectrum of agriculturally important bacteria and fungi including both monocot and dicot pathogens with diverse infection strategies, were assessed for antimicrobial activity of the culture filtrate (CF) supernatant and secondary metabolites from strain JCK-8368 using the broth dilution method (Le et al., 2021). The minimum inhibitory concentration (MIC) is the lowest concentration that inhibits microorganism growth. Each experiment included three replicates and was repeated twice. Stretomycin sulfate (200 µg/mL) was used as a positive control. To evaluate antibacterial activity, the following phytopathogens were used: *Acidovorax avenae* subsp. *cattleyae*, *Acidovorax konjaci*, *Pectobacterium carotovorum* subsp. *carotovorum*, *Pseudomonas syringae* pv. *actinidiae*, *Pseudomonas syringae* pv. *lachrymans*, *Ralstonia solanacearum* SL341, *Xanthomonas arboricola* pv. *pruni*, and *Erwinia amylovora* TS3128. These strains were obtained from the Rural Development Administration, Dong-A University, Suncheon National University, and the Korea Research Institute of Chemical Technology. To evaluate antifungal activity, the phytopathogenic fungi *Botryosphaeria dothidea, Botrytis cinerea, Clarireedia homoeocarpa*, and *Rhizoctonia solani* AG 2–2 were obtained from the Korea Research Institute of Chemical Technology.

### 2.4 Histochemical analysis of *β-glucuronidase* activity

The seeds of transgenic *Arabidopsis thaliana* carrying the pathogenesis-related 1 (*PR1)* promoter fused to the *β-glucuronidase* (GUS) were used to study the expression of GUS (Park et al., 2020). They were sterilized and seeded following the protocol outlined by Nguyen et al. (2024). The culture broth (CB) and secondary metabolites of JCK-8368 were then assessed using the GUS assay. JCK-8368 was incubated in tryptic soy broth (TSB) at 28°C and 180 rpm for 7 days. The CB was then separated into CF and cell suspension (CS) and diluted at 250-fold, 500-fold, and 1,000-fold dilutions. Samples were labeled as: CBA400, CFA400, CSA400 for the 250-fold dilution; CBA200, CFA200, and CSA200 for the 500-fold dilution; and CBA100, CFA100, and CSA100 for 1,000-fold dilution. The secondary metabolite was dissolved in acetone at 2 mg/mL, then diluted with sterile distilled water (SDW) to 1,000 ng/mL, 100 ng/mL, 20 ng/mL, 10 ng/mL, and 1 ng/mL labeled as A1000, A100, A20, A10, and A1, respectively. These samples were individually applied to *A. thaliana* seedlings and incubated at 25°C for 48h. After treatment, the seedlings were stained with a chemical solution (Kondo et al., 2014). SA served as the positive control, whereas TSB, 1% acetone and SDW were the negative controls. Each trial comprised three replicates and was repeated twice.

### 2.5 *In* planta bioassays

#### 2.5.1 Efficacy of secondary metabolites and culture filtrate supernatants JCK-8368 against tomato bacterial wilt

The antibacterial efficacy of JCK-8368 against tomato bacterial wilt was assessed using a wettable powder formulation containing its secondary metabolite, The wettable powder formulation of ethyl acetate extract (EtOAc) of JCK-8368 (named EWP10) was prepared as follows: The EtOAc extract were mixed with synthesized hydrated silicon dioxide (white carbon; Rhodia Asia Pacific Pte Ltd., Kallang, Singapore), sodium dodecyl sulfate, (CR-SDS; Yoosung Chemical R&T Co., Ltd., Chungnam, Republic of Korea), sodium poly (naphthalene formaldehyde) sulfonate (CR-100; Yoosung Chemical R&T Co., Ltd., Chungnam, Republic of Korea), and kaoline to create WP-type formulations. Briefly, 1g of the EtOAc extract was mixed with 1.5g of silicon dioxide, 0.5g of sodium dodecyl sulfate, 0.5g of sodium poly (naphthalene formaldehyde) sulfonate, and 6.5g of kaoline to create the EWP10. The formulations were finely mixed in a blender (Chung et al., 2023). The EWP10 formulation was treated at a 1,000-fold dilution. A 1,000-fold dilution of Seongbocycline (oxytetracycline 17% WP, Sungbo Chemicals Co., Ltd., Gyeonggi, Republic of Korea) served as the standard control. All samples were diluted with SDW, which also served as the untreated control. In total, 20 mL of each sample was drained from the soil 1 day before inoculation (DBI). The experiment was conducted in triplicate with three replications.

The potential of the CF supernatant of JCK-8368 and its secondary metabolite to induce resistance against *R. solanacearum* in tomatoes was evaluated at low concentrations, including CFA400, CFA200, CFA100, A1000, A100, A20, A10, and A1. All samples were diluted with SDW and supplemented with 250 μg/mL of Tween 20 (Sigma-Aldrich, St. Louis, MO, USA). SDW mixed with methanol at 1% and Tween 20 at 250 μg/mL was used as an untreated control. CF supernatants were applied either by soil drenching (20 mL/plant) or foliar spraying (8 mL/plant), whereas compound solutions were administered merely via soil drenching (20 mL/plant). Treatments were applied at 3 DBI. The experiment was performed in triplicate and repeated three times.

The experiments were conducted at the fourth-leaf stage of Seokwang tomato seedlings (FarmHannong Co., Ltd, Seoul, Republic of Korea). The plants were inoculated with a suspension of *R. solanacearum* SL341 at a concentration of 10^8^ colony-forming units (CFU)/mL through soil drenching and maintained at 30 ± 2°C with 75% humidity under a 12-h photoperiod. The pathogenic inoculation and symptoms of tomato bacterial wilt or disease severity (DS) were conducted at 7 days after inoculation (DAI) following the methods of Vu et al. (2017) and Nguyen et al. (2024).

#### 2.5.2 Efficacy of wettable formulation and culture filtrate supernatant of JCK-8368 against apple fire blight

The antibacterial efficacy of JCK-8368 against apple fire blight was evaluated using EWP10. Briefly, apple seedlings were foliar-sprayed with a 500-fold dilution of EWP to assess its antibacterial efficacy. Commercial bactericide Agrepto (streptomycin 20%; Kyung Nong, Seoul, Republic of Korea) served as the positive control. All samples were diluted with SDW, which also served as the untreated control. Approximately 8 mL of each sample was foliar-sprayed at 1 DBI. The experiments were performed in triplicate and repeated twice.

The potential induced resistance of JCK-8368 against apple fire blight was assessed using CFA100. CFA100 was added with Tween 20 (Sigma-Aldrich, St. Louis, MO, USA) at 250 μg/mL. Serifel (containing *Bacillus amyloliquefacciens* subsp. *plantarum* MBI600; BASF, Seoul, Republic of Korea) served as the positive control. All samples were diluted with SDW. SDW containing 250 μg/mL of Tween 20 served as the untreated control. Approximately 8 mL of each sample was foliar-sprayed twice at 10 and 3 DBI. The experiments were performed in triplicate and repeated twice.

M9 apple seedlings (Korea Technology Promotion Agency, Iksan, Republic of Korea) measuring 15 ± 3cm in height were used. The leaves were sprayed with 10 mL of bacterial phytopathogenic strain *E. amylovora* TS3128 suspension (3.3 × 10^7^ CFU/mL) and kept moist by covering them with plastic bags for 2 days. The temperature was maintained at 25°C for 14 days. Fire blight symptoms were assessed and rated at 7, 10, and 14 DAI using the DS index (Hevesi et al., 2000; Nguyen et al., 2024).

The control value was calculated using the following equation:


$$Control\;value\;(\%)=\frac{(DS\;of\;untreated\;control\;-\;DS\;of\;treatment)\;}{DS\;of\;untreated\;control}\times 100\%$$

where DS of untreated control is the average value of disease severity of untreated pots, and DS of treatment is the average value of disease severity of treated pots.

### 2.6 RNA isolation and quantitative real-time polymerase chain reaction

RNA extraction and cDNA synthesis were performed following Nguyen et al. (2024). The experiment was conducted on apple and tomato seedlings. Briefly, the M9 apple seedlings were foliar-sprayed with CFA100 at 10 and 3 DBI with *E. amylovora* TS3128. The Seokwang tomato seedlings were soil-drenched with A100 at 3 DBI with *R. solanacearum* SL341. Inoculation of *E. amylovora* TS3128 on apple seedlings and *R. solanacearum* SL341 on tomato seedlings followed the protocols outlined in the previously described *planta* bioassay. The leaves from three plants per groups were individually harvested for RNA extraction at 0, 1, 2, and 3 DAI. qRT-PCR was performed with three technical replicates for each of the three biological samples. **Supplementary Table S1** provides a list of the primers. The relative expression of the target genes was determined using the method of Livak and Schmittgen (2001). The defense genes were selected based on their association with either the salicylic acid (SA) or jasmonic acid (JA) signaling pathways, as well as the availability of their primers.

### 2.7 Statistical analysis

The pot experiment results were statistically analyzed using SPSS Statistics software version 20.0 (IBM Corp., Armonk, NY, USA). Student’s t-test, one-way, and two-way analysis of variance were performed, followed by Tukey’s honestly significant difference test. The results of the replicates were shown as the mean ± standard error (bars). Graphs were generated using GraphPad Prism 8.0 software (GraphPad Software, Inc., La Jolla, CA, USA).

## 3 Results

### 3.1 Phenotypic and phylogenetic features of JCK-8368

**Figure 1:**
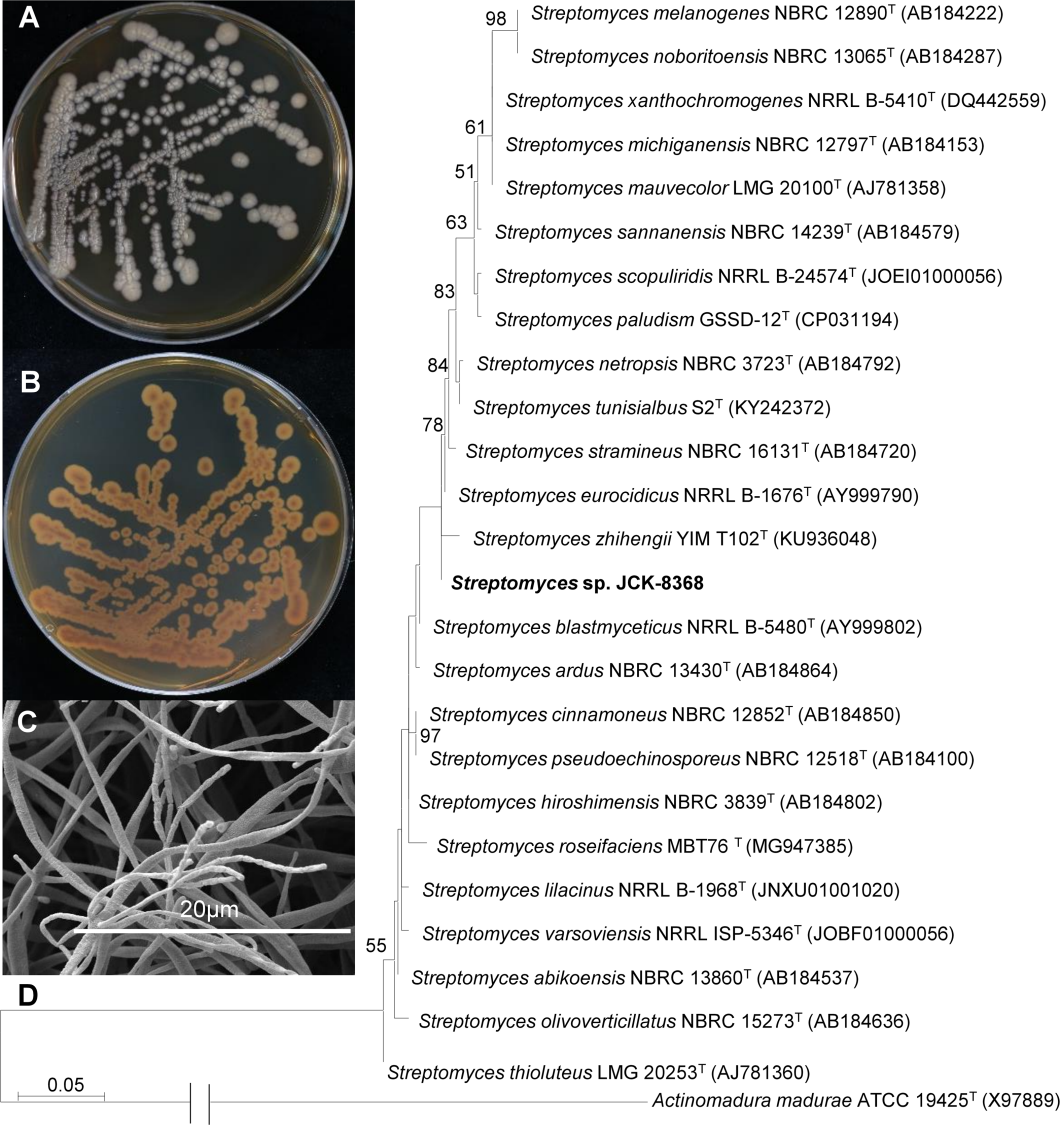
Growth and morphology of JCK-8368 on TSA plates. **(A)** Top view and **(B)** bottom view of TSA plates showing the growth of JCK-8368 colonies after 7 days of incubation at 28°C. **(C)** Spore chain morphology of JCK-8368 assessed using SEM. **(D)** Maximum-likelihood phylogenetic tree based on the 16S rRNA sequences of JCK-8368 and related *Streptomyces* species. *Actinomadura madurae* ATCC 19425^T^ was used as the outgroup. Bootstrap values (≥ 50%) based on 1,000 replicates are shown at branch nodes. The scale bar indicates 0.05 nucleotide substitutions per nucleotide position. TSA, tryptic soybean agar; SEM, scanning electron microscope; rRNA, ribosome ribonucleic acid.

The strain grew on TSA plate and produced a pale brown, soluble pigment (**Figures 1A, B**). It developed sporulating mycelia, with spore chains exhibiting an umbellate monoverticilate morphology (**Figure 1C**). The 16S rRNA gene sequence of JCK-8368 can be found under accession number OR130923 in the GenBank/EMBL/DDBJ databases. This sequence exhibited the highest similarity to that of *S. albireticuli* NBRC 12737^T^ (99.92%) and *S. eurocidicus* NBRC 13491^T^ (99.85%). The neighbor-joining phylogenetic tree revealed that the strain was affiliated with the genus *Streptomyces*, forming a monophyletic clade with *S. eurocidicus* NBRC 13491^T^ (**Figure 1D**). Overall, the phenotypic and phylogenetic characteristics supported the classification of JCK-8368 as a member of the genus *Streptomyces*.

### 3.2 Isolation and identification of the bioactive metabolite

**Figure 2:**
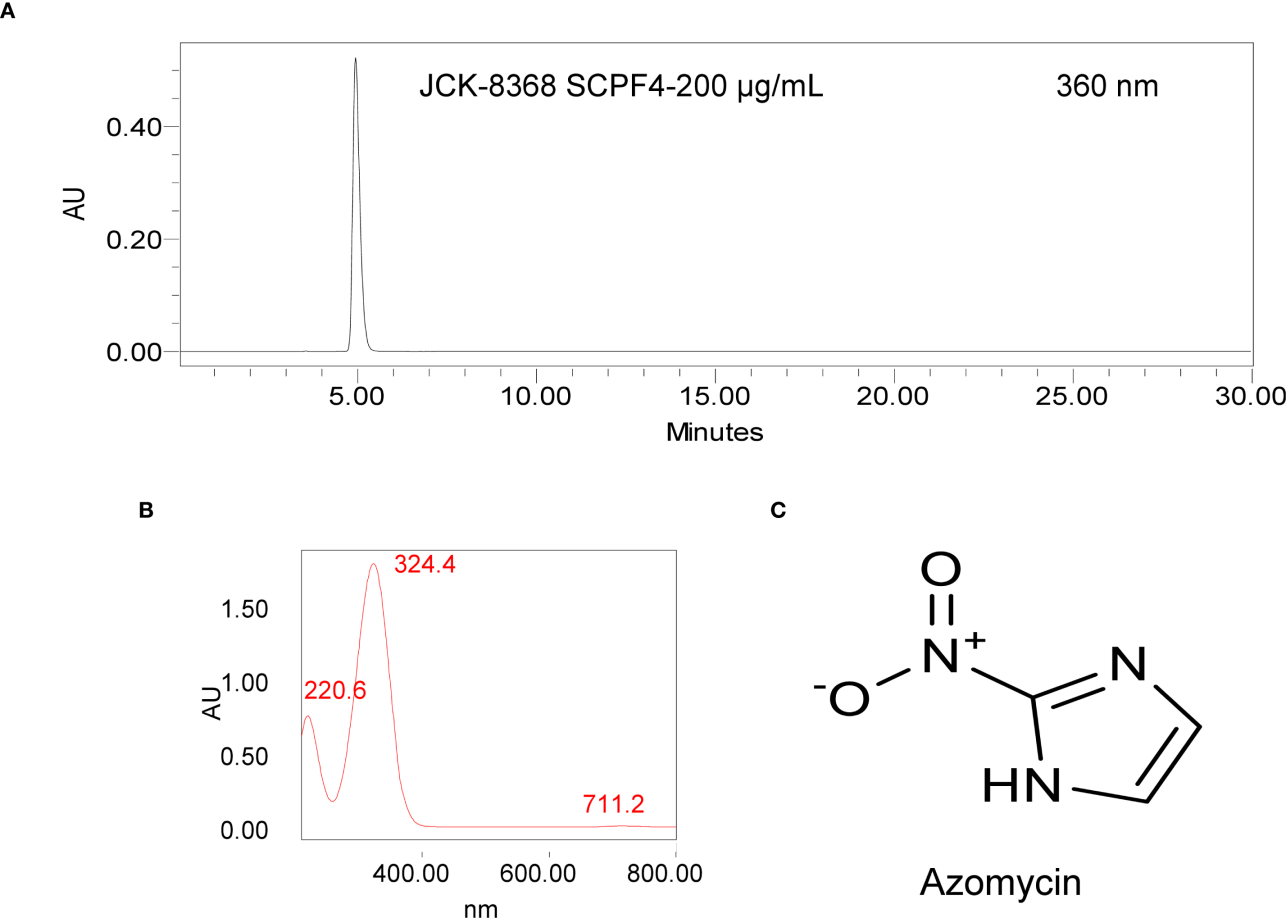
HPLC chromatogram and chemical structure of the antibacterial metabolite produced by JCK-8368. **(A, B)** HPLC chromatogram and UV spectra of the isolated fraction, **(C)** chemical structure of azomycin. HPLC, high-performance liquid chromatography.

Among the two solvent layers and the aqueous layer, only the EtOAc layer exhibited antibacterial activity against *E. amylovora* TS3128. To isolate bioactive metabolites, multiple rounds of column chromatography and preparative TLC were performed on the EtOAc layer, guided by an antibacterial bioassay against *E. amylovora* TS3128. This process yielded 23.5 mg of a colorless crystalline compound, designated SCPF4 (**Supplementary Figure S1**). The compound exhibited a single peak on the HPLC chromatogram, confirming its purity (**Figure 2A**). The UV spectrum of the compound exhibited two distinct maxima peaks at approximately 220.6 nm and 324.4 nm (**Figure 2B**), indicating a similarity to that of 2-nitroimidazole. UHPLC-Q-Orbitrap MS analysis of SCPF4 in negative ion mode revealed protonated molecular ions [M – H]^−^ at *m/z* 112.01 (**Supplementary Figure S2**), indicating a molecular formula of C_3_H_2_N_3_O_2_^-^. GC/MS analysis revealed the molecular mass of this compound at a molecular peak at *m/z* 113 in the HESI-MS spectrum, and library search was indicative of 4-nitroimidazole (**Supplementary Figure S3**). Furthermore, the ^1^H-NMR spectrum of this compound shows a symmetrical structure with a singlet at δ 7.3 ppm for 2 aromatic protons (at C4 and C5) (**Supplementary Figure S4**). The ^1^H-NMR characteristic of this compound differed from 4-nitroimidazole’s, which displays two separate proton signals in a range of 7.8 to 8.2 ppm (Orsi et al., 2017; Backler et al., 2020). Collectively, SCPF4 was identified as 2-nitroimidazole (azomycin, C_3_H_3_N_3_O_2_) (**Figure 2C**), an isomer of 4-nitroimidazole (Orsi et al., 2017). HPLC analysis of the JCK-8368 CF supernatant revealed a productivity value of 94.15 ± 0.08 µg/mL of azomycin (**Supplementary Figure S5**).

### 3.3 *In vitro* antibacterial efficacy against phytopathogens

The JCK-8368 culture filtrate exhibited antimicrobial activity against various phytopathogens, including *A. konjaci*, *E. amylovora* TS3128, *R. solanacearum* SL341, *X. arboricola* pv*. pruni*, *C. homoeocarpa*, and *R. solani* AG 2-2 (**Table 1**, **Supplementary Table S2**). The bioactive compound azomycin exhibited antibacterial and antifungal activity against all tested microorganisms. It completely inhibited eight tested bacterial strains, with MIC values ranging from 0.65 to 200 µg/mL (**Table 1**) and suppressed fungal growth with MIC values from 6.25 to 200 µg/mL (**Supplementary Table S2**). *R. solanacearum* SL341 and *E. amylovora* TS3128 were highly sensitive to azomycin, with MIC values of 3.12 ± 0.00 µg/mL and 20.83 ± 6.36 µg/mL, respectively, compared to MIC values of 2.08 ± 0.30 µg/mL and 3.12 ± 0.00 µg/mL for streptomycin sulfate.

**Table 1:** MICs of the fermentation filtrate obtained from *Streptomyces* sp. JCK-8368 and its antibacterial metabolite against phytopathogenic bacteria.

Phytopathogenic bacteria	MIC		
	Fermentation filtrate (%)	Azomycin (µg/mL)	Streptomycin sulfate (µg/mL)
*Acidovorax avenae* subsp. *cattleyae*	> 10.00 ± 0.00	100.00 ± 0.00	> 200.00 ± 0.00
*Acidovorax konjaci*	10.00 ± 0.00	33.33 ± 12.73	12.50 ± 0.00
*Erwinia amylovora* TS3128	10.00 ± 0.00	20.83 ± 6.36	3.12 ± 0.00
*Pectobacterium carotovorum* subsp. *Carotovorum*	>10.00 ± 0.00	100.00 ± 0.00	10.42 ± 1.20
*Pseudomonas syringae* pv. *actinidiae*	> 10.00 ± 0.00	200.00 ± 0.00	2.60 ± 0.79
*Pseudomonas syringae* pv. *Lachrymans*	> 10.00 ± 0.00	100.00 ± 0.00	> 200.00 ± 0.00
*Ralstonia solanacearum* SL341	10.00 ± 0.00	3.12 ± 0.00	2.08 ± 0.30
*Xanthomonas arboricola* pv*. pruni*	0.41 ± 0.16	0.65 ± 0.20	8.33 ± 3.18

Data are expressed as mean ± standard deviation of three replicates.

MICs, Minimum inhibitory concentrations.

### 3.4 β-glucuronidase staining assay

**Figure 3:**
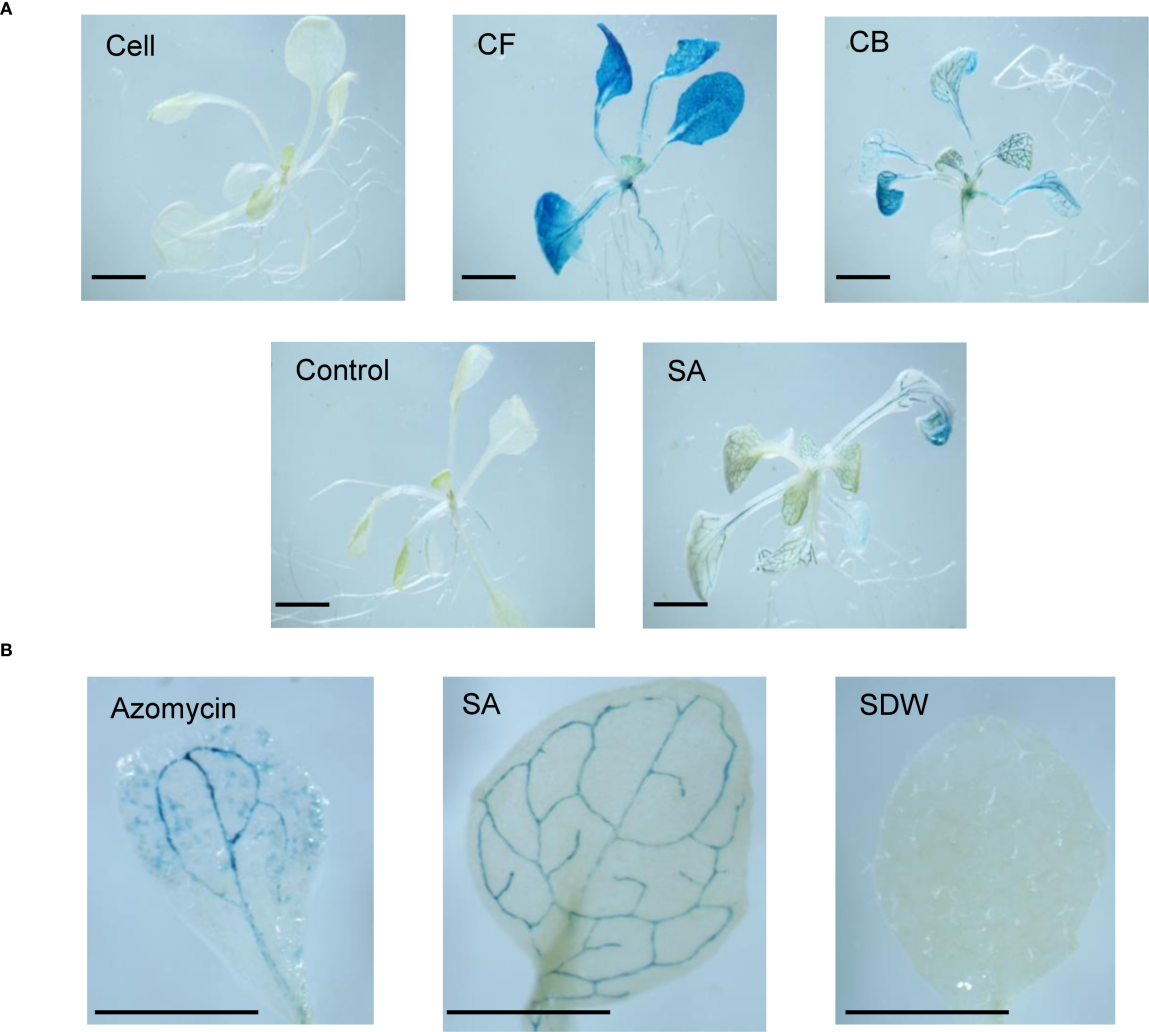
GUS activity in transgenic *Arabidopsis thaliana* rosette leaves in response to treatment with **(A)** 1,000-fold dilution of JCK-8368 fermentation broth and **(B)** azomycin (100 ng/mL). CFA100, culture filtrate; CBA100, culture broth; CSA100, cell suspension, control, 0.05% TSB; SA, salicylic acid (0.1 mM); SDW, sterile distilled water; GUS, β-glucuronidase; TSB, tryptic soybean broth. Scale bar, 1.0mm.

SA treatment induced GUS activity, indicated by blue staining in the vascular tissues of leaves, stems, and roots. *A. thaliana* seedlings treated with the CB and CF of JCK-8368 exhibited similar GUS activity to that of SA-treated seedlings, displaying blue coloration on 4–5 leaves or all leaves. However, seedlings treated with the cells or TSB showed no activity (**Figure 3A**). All azomycin treatments also elicited positive responses on a single leaf, though the staining was weaker than induced by JCK-8368 (**Figure 3B**, **Supplementary Figure S6**).

### 3.5 Efficacy of JCK-8368 against tomato bacterial wilt and apple fire blight disease

#### 3.5.1 Tomato bacterial wilt

Tomato bacterial wilt was introduced through the soil, and EWP10 and Seongbocycline were applied via soil drenching. EWP was treated at a 1,000-fold dilution equivalent to 4.7 µg/mL of azomycin and exhibited protective activity against *R. solanacearum* SL341. It achieved a 100.00% control value, significantly higher than that of Seongbocycline, with approximately 57.57% control against *R. solanacearum* SL341 at the tested concentration (*p <*0.05) (**Supplementary Figure S7**). Young leaves treated with EWP10 at 1,000-fold dilution initially turned yellow but later recovered. (**Supplementary Figure S7**).

**Figure 4:**
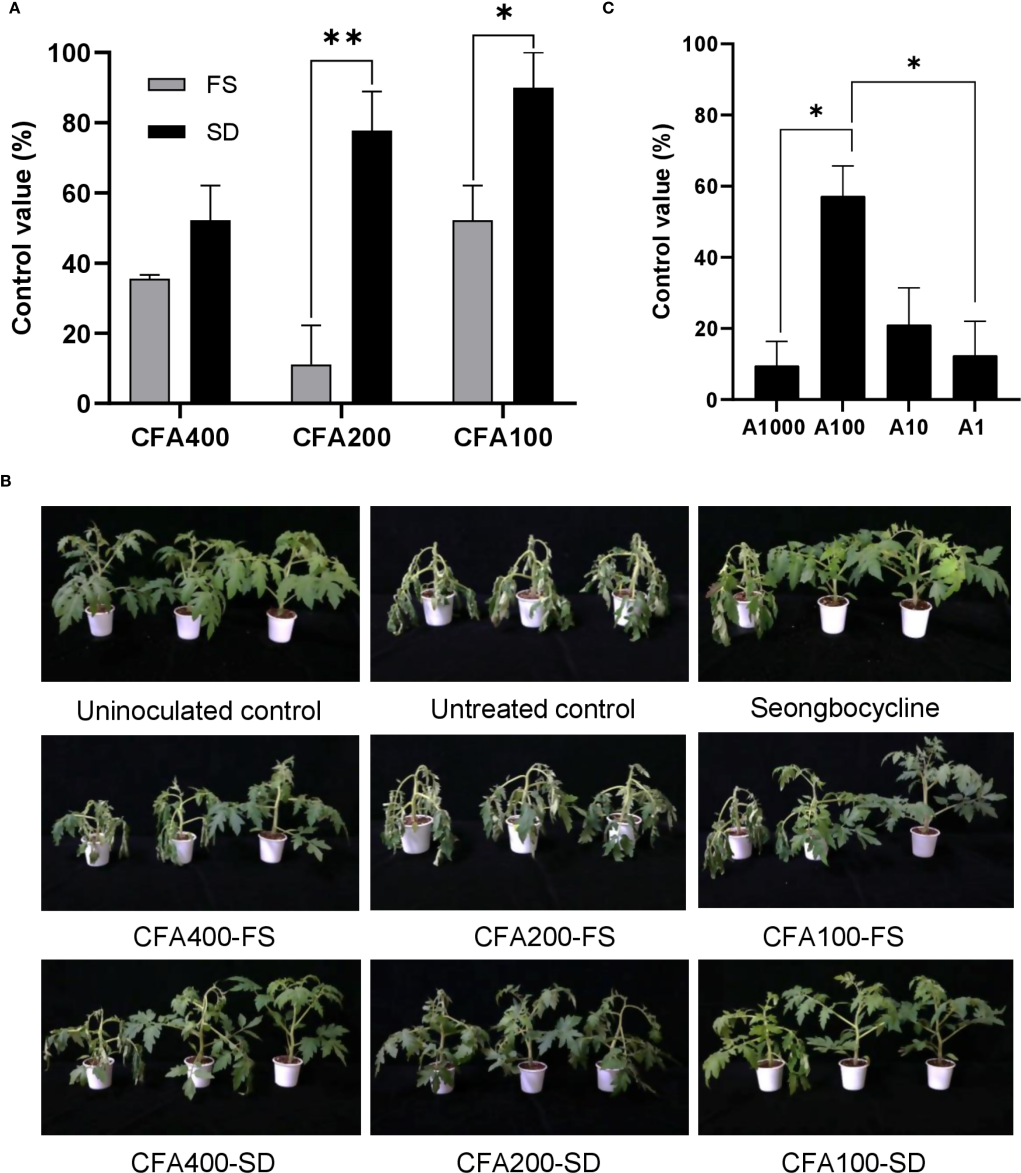
Disease control efficacy of pretreatment with JCK-8368 **(A, B)** and its secondary metabolite **(C)** against tomato bacterial wilt caused by *R. solanacearum* SL341. CFA400, culture filtrate of JCK-8368 at 250-fold dilution; CFA200, culture filtrate of JCK-8368 at 500-fold dilution; CFA100, culture filtrate of JCK-8368 at 1000-fold dilution; FS, foliar spray; SD, soil drench; A1,000, azomycin 1,000 ng/mL; A100, azomycin 100 ng/mL; A10, azomycin 10 ng/mL; A1, azomycin 1 ng/mL. Error bars indicate standard errors. *(*p <*0.05) and **(*p <*0.01) represent significant differences by the Tukey’s HSD test. HSD, honestly significant difference.

Since tomato bacterial wilt is a soilborne disease, foliar spraying was expected to be ineffective. However, the application of CFA400 through foliar spraying and soil drenching resulted in similar disease control rates (35.55% and 52.22%, respectively), with no significant difference observed (*p* > 0.05) (**Figure 4A**). The CFA200 and CFA100 exhibited greater efficacy against *R. solanacearum* SL341 when applied through soil drenching (77.78% and 90.00% control values, respectively) than when applied through foliar spraying (11.11% and 52.22% control values, respectively) (p < 0.01 and *p <*0.05, respectively). Soil-drenching treatments exhibited an inverse dose-dependent response, with the highest control efficacy observed at CFA100. Additionally, at this lowest CF concentration, foliar spraying also effectively inhibited disease progression with a control efficacy of 52.22%. No phytotoxic symptom was observed in any treated sample (**Figure 4B**). The CF supernatant contained 94.15 ± 0.08 µg/mL of azomycin (**Supplementary Figure S5**), indicating that the CF samples including CFA400, CFA200, and CFA100 correspond to 400 ng/mL, 200 ng/mL, and 100 ng/mL of azomycin, respectively.

The secondary metabolite, azomycin, inhibited disease development at all tested concentrations (1, 10, 100, and 1,000 ng/mL, corresponding to A1, A10, A100, and A1,000), with control values significantly higher that of untreated samples (*p <*0.05) (**Figure 4C**). Among them, A100 most effectively suppressed the development of tomato bacterial wilt with a control value of 57.14% (*p <*0.05). Azomycin displayed control efficacy in a dose-dependent manner between A1 and A100 but showed significantly reduced efficacy at A1000. None of the samples exhibited phytotoxicity (**Supplementary Figure S8**).

#### 3.5.2 Apple fire blight

Since apple fire blight is an airborne disease, EWP and Agrepto were treated via foliar spray. EWP10 was treated at a 500-fold dilution equivalent to 8.4 µg/mL of azomycin. It effectively inhibited the spread of apple fire blight at 14 DAI (70.00%) (**Supplementary Figure S9**). Agrepto also demonstrated complete disease control efficacy with a 100.00% control value. Yellow coloration appeared along the veins of apple leaves treated with Agrepto. No phytotoxic symptoms were observed in the other samples (**Supplementary Figure S9**).

**Figure 5:**
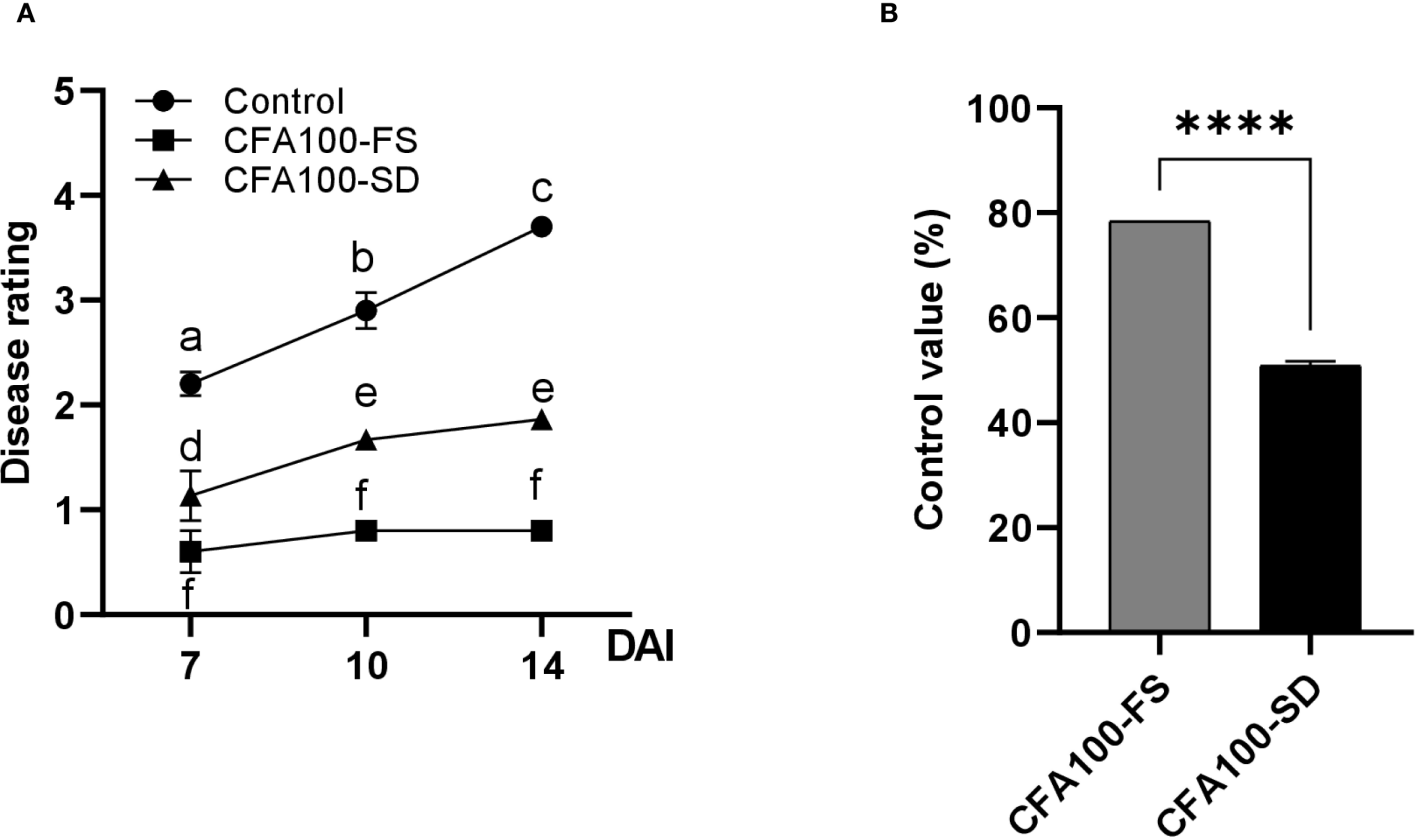
Disease control efficacy of JCK-8368 against apple fire blight caused by *E*. *amylovora* TS3128. **(A)** Disease progression in the treatment groups, measured by the disease rating of M9 apple plants inoculated with *E. amylovora* TS3128. **(B)** Efficacy of JCK-8368 pretreatment via foliar spray and soil drenching against apple fire blight. CFA100, culture filtrate of JCK-8368 at 1,000-fold dilution; FS, foliar spray; SD, soil drench; DAI, day after inoculation. Error bars indicate standard errors. Different letters and ****(*p <*0.0001) represent significant differences by the Tukey’s HSD test. HSD, honestly significant difference.

As apple fire blight is an airborne disease, soil drenching was expected to be ineffective in preventing its development. In the *in vivo* experiment, the DS of untreated apples steadily increased, reaching 2.2, 2.9, and 3.7 at 7, 10, and 14 DAI, respectively. In contrast, the DS of apples treated with CFA100 slightly increased or remained unchanged throughout the period, regardless of applying soil drenching or foliar spraying. The DS values were 0.60, 0.80, and 0.80 after foliar spraying and 1.13, 1.67, and 1.87 after soil drenching at 7, 10, and 14 DAI, respectively (**Figure 5A**). Furthermore, CFA100 suppressed disease development at 14 DAI regardless of treatment method. The control values for foliar spraying (78.38%) exceeded those of soil drenching (50.88%) (*p <*0.0001) (**Figure 5B**).

### 3.6 Time-dependent expression of differential genes

**Figure 6:**
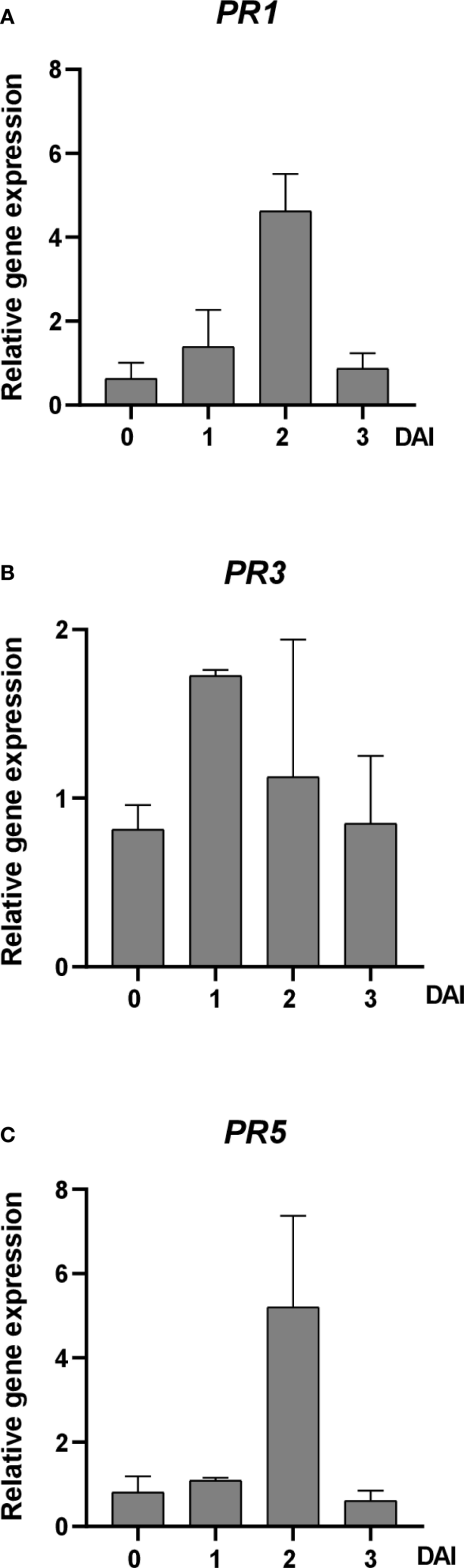
Effect of *Streptomyces* sp. JCK-8368 culture filtrate on gene expression levels in apple plants before and after inoculation with *E*. *amylovora*. The expression levels of **(A)** *PR1*, **(B)** *PR3*, and **(C)** *PR5* were analyzed in plants sprayed with 0.5% tryptic soybean broth (untreated) or treated with CFA100 (1,000-fold-diluted culture filtrate) at different times, including 0, 1, 2, and 3 DAI. Error bars indicate standard errors. DAI, days after inoculation; PR, pathogenesis-related.

The expression patterns of defense-related genes, including *PR1*, *PR3*, and *PR5*, were examined in apple seedlings after CFA100 treatment and subsequent inoculation with *E. amylovora* TS3128. At 0 DAI, no significant changes occurred in gene expression. However, at 1 DAI, *PR1* and *PR3* expression slightly increased by 1.39-fold and 1.73-fold, respectively, compared to those in the control plants. At 2 DAI, *PR1* and *PR5* expression were upregulated by 4.63-fold and 5.21-fold, respectively. Subsequently, at 3 DAI, the expression levels of all three genes decreased (**Figure 6**).

**Figure 7:**
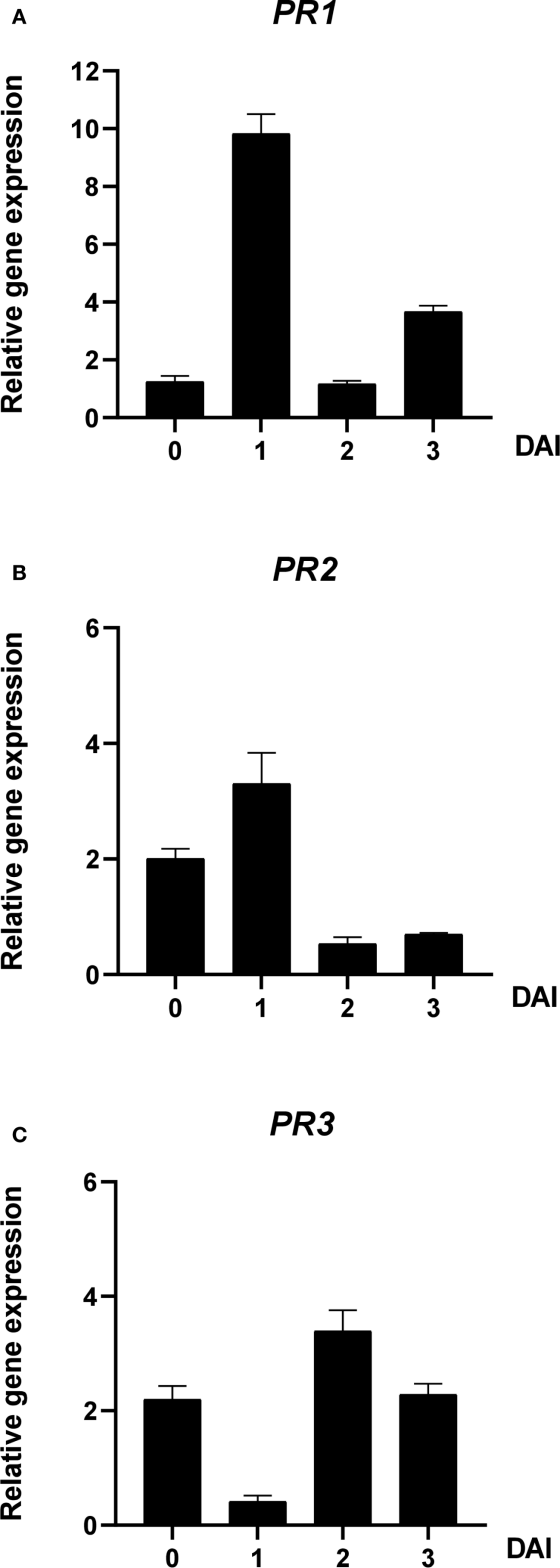
Effect of A100 (azomycin at 100 ng/mL) on gene expression levels in tomato seedlings before and after inoculation with *R. solanacerum* SL341. The expression levels of **(A)** *PR1*, **(B)** *PR2*, and **(C)** *PR3* were analyzed in plants treated with 1% acetone plus 250 µg/mL of Tween 20 (untreated), including 0, 1, 2, and 3 DAI. Error bars indicate standard errors. DAI, days after inoculation; PR, pathogenesis-related.

The expression patterns of defense-related genes *PR1*, *PR2*, and *PR3* in tomato seedlings were assessed after A100 treatment and inoculation with *R. solanacearum* SL341. At 0 DAI, *PR1*, *PR2*, and *PR3* expression upregulated by 1.25-fold, 2.00-fold and 2.20-fold, respectively. However, at 1 DAI, *PR1* and *PR2* expression increased by 9.82-fold and 3.30-fold, respectively, while *PR3* expression decreased to 0.41-fold. At 2 DAI, *PR1* and *PR2* expression dropped by 1.17-fold and 0.53-fold, respectively, whereas *PR3* increased by 3.39-fold. At 3 DAI, *PR1* and *PR2* expression increased by 3.67-fold and 0.70-fold, respectively, and *PR3* declined to 2.28-fold (**Figure 7**).

## 4 Discussion

Bacterial wilt and fire blight, caused by *R. solanacearum* and *E. amylovora*, respectively, rank among the top 10 economically and scientifically significant bacterial plant diseases (Mansfield et al., 2012). *R. solanacearum* is a soilborne phytopathogen that infects over 310 plant species and survives for long periods in the environment (Álvarez et al., 2019; Genin, 2010; Genin and Denny, 2012). It invades the root xylem and infects plant roots via small wounds, rapidly spreading to the stem tissue (Wang et al., 2023a). *E. amylovora* is an airborne pathogen that colonizes most species within the subfamily Maloideae of the family Rosaceae (Hildebrand et al., 2001; Paulin, 2000). During colonization, this bacterium initially replicates on the surface of the stigma, then moves down the flower style, aided by precipitation or heavy dew (Bubán and Orosz-Kovács, 2003; Thomson, 1986). Although multiple methods have been developed for disease management, effective strategies that combine eco-friendliness and control remain necessary. *Streptomyces* strains show potential as biocontrol agents against phytopathogens. For instance, *S. lydicus* WYEC108 has been used to control *Fusarium* spp. and *Pythium* spp. in the soil, while *S. griseoviridis* K61 effectively suppresses *Botrytis cineria* and *Phytophthora* spp (Rey and Dumas, 2017). The biocontrol efficacy of these *Streptomyces* species primarily stems from their antimicrobial activity (Vurukonda et al., 2018). Recent studies report that *Streptomyces* spp. JCK-6311 and JCK-8055 control plant diseases through direct antimicrobial and induced resistance mechanisms (Gang et al., 2019; Le et al., 2021; Nguyen et al., 2024) However, the specific secondary metabolites responsible for these effects remained unidentified (Le et al., 2021; Nguyen et al., 2024). In this study, *Streptomyces* sp. JCK-8368 demonstrated strong potential to control tomato bacterial wilt and apple fire blight through antimicrobial activity and activation of plant defense responses, mainly via azomycin production.

*Streptomyces* is the largest genus known for producing numerous antibiotics (Rey and Dumas, 2017). These bacteria are often isolated from plant root environment (Schrey and Tarkka, 2008). In our study, the *Streptomyces* sp. JCK-8368 was isolated from the root of chilli plant and its culture filtrate demonstrated antimicrobial activity against *R. solanacearum* and *E. amylovora*. This finding suggests that JCK-8368 effectively suppresses these phytopathogenic bacteria by producing antimicrobial compounds. To identify the specific metabolites responsible for the antibacterial effects, UPLC-MS, GC-MS, and NMR analyses were performed to elucidate their chemical structures. The metabolite produced by JCK-8368 was identified as 2-nitroimidazole (azomycin), a symmetrical member of the nitroimidazole class of nitroheterocyclic compounds (Ang et al., 2017; Rashed et al., 2022). It was originally isolated from *Streptomyces* sp. and named azomycin (Rashed et al., 2022). Azomycin exhibits broad-spectrum antimicrobial activity against human pathogens, attributed to the nitro group in its chemical structure (Ang et al., 2017). The nitro group undergoes reduction, generating reactive radical species that interact with cellular components, such as DNA or proteins (Ang et al., 2017). These reactive intermediates induce DNA damage, leading to bacterial cell death (Crişan et al., 2024; Edwards, 1993a, b). In this study, azomycin exhibited antibacterial and antifungal activities against various selected phytopathogenic bacteria and fungi, suggesting its strong potential for plant disease management. To our knowledge, this is the first study to report the growth-inhibiting capabilities of azomycin against phytopathogens, such as *E. amylovora* and *R. solanacearum*.

Azomycin exhibited antibacterial activity against *E. amylovora* TS3128 and *R. solanacearum* SL341, with MIC values of 20.83 µg/mL and 3.12 µg/mL, respectively. In the *in vivo* experiment, treatment with a wettable powder formulation of ethyl acetate (EWP10) containing 9.4 µg/mL or 4.7 µg/mL of azomycin significantly suppressed the development of apple fire blight and tomato bacterial wilt, achieving control values of 70.00% and 100.00%, respectively. However, phytotoxic symptoms or stress-inducing effect were observed on tomato plants at this concentration. These findings suggest that azomycin produced by *Streptomyces* sp. JCK-8368 effectively suppresses plant bacterial diseases through direct antibacterial activity. Redox-active compounds modulate plant defense mechanisms by influencing reactive oxygen species levels or activating redox-sensitive genes (Gonzalez-Bosch, 2018; Klessig et al., 2000; Nawrocka et al., 2019). For example, glutathione, a redox-active molecule, plays a key role in maintaining cellular redox homeostasis and activating plant defense responses (Kovacs et al., 2015). Based on this, we hypothesized that the reductive activation of azomycin may also induce moderate stress in the plant tissue, potentially triggering defense priming mechanisms that enhance resistance to subsequent biotic stress. However, considering its potential phytotoxicity, further studies on optimal application concentrations and delivery methods are necessary to maximize its efficacy while minimizing any phytotoxic risks.

*Streptomyces* strains and their metabolites effectively suppress phytopathogens through direct antimicrobial activity and induce plant resistance (Shepherdson et al., 2023). *Streptomyces* strains effectively control tomato bacterial wilt at low CF concentrations by inducing resistance mechanisms (Le et al., 2021). Consistently, we found that low concentrations of JCK-8368 CF strongly inhibit the development of plant bacterial diseases. It demonstrated a reversed dose-dependent effect in controlling tomato bacterial wilt. Among tested concentrations, CFA100—the lowest CF—showed the highest efficacy (up to 90.00%) in controlling tomato bacterial wilt when applied through soil drenching. In addition, CFA100 suppressed the disease by 52.23% through foliar spraying. Furthermore, foliar spraying and soil drenching with CFA100 inhibited the development of apple fire blight by 78.38% and 50.88%, respectively. Therefore, CFA100 suppresses the development of these diseases with control values above 50%, regardless of soil drenching or foliar spraying. These findings highlight the effectiveness and potential of JCK-8368 in managing plant diseases. Moreover, applying low concentrations of azomycin, ranging from 1 ng/mL to 1,000 ng/mL (A1 to A1,000), through soil drenching exhibited a dose-dependent response between 1 and 100 ng/mL, which significantly reduced at 1000 ng/mL. The highest control value of 57.14% occurred at 100 ng/mL (A100), equivalent to CFA100. At this concentration, JCK-8368 CF also achieved its highest control value against tomato bacterial wilt in the induced resistance assay. At elevated concentrations, certain metabolites can become phytotoxic or stress-inducing, potentially impairing the host plant’s defense capacity. For example, excessive levels may also disrupt plant–microbe signaling, downregulating induced resistance pathways. In contrast, lower concentrations may better mimic natural microbe–plant interactions, promoting optimal systemic resistance without causing physiological stress. Similar trends have been reported in plant–microbe systems where metabolite overaccumulation suppresses brather than enhances immunity (Prithiviraj et al., 2007). In this study, both azomycin (EWP10) and streptomycin sulfate (Agrepto) effectively control bacterial plant diseases, but high concentrations can induce phytotoxicity. Streptomycin sulfate, although widely used against fire blight, has been reported to cause persistent leaf yellowing in apple (*Malus* spp.) when overapplied (Schreiber et al., 1981). In our experiemnt, high concentrations of azomycin, 1,000-fold dilution of EWP10, caused visible leaf yellowing in tomato, likely due to oxidative stress or interference with chlorophyll biosynthesis (Wang et al., 2023b). Unlike streptomycin-induced injury, azomycin symptoms were transient, with plants recovering normal coloration, suggesting reversible stress. Dosage optimization and delivery method strongly influenced safety and efficacy—soil drenching at moderate doses caused fewer symptoms and greater control than equivalent foliar applications. Strategies such as lowering concentrations with increased frequency, controlled-release formulations, or protective adjuvants may further improve azomycin’s safety profile while maintaining disease control. In addition, CFA100, containing 100 ng/mL of azomycin, exhibited a control efficacy similar to EWP10 treatment, which contained 9.4 µg/mL of azomycin, achieving control values of 78.38% and 70.00%, respectively, against apple fire blight. These findings further led us to hypothesize that JCK-8368 and azomycin not only directly combat pathogens but also induce and strengthen plant defense, with azomycin likely serving as the key active compound of JCK-8368. Our hypothesis was supported by the GUS assay revealing that the JCK-8368 CF supernatant and azomycin induced GUS activity in transgenic *Arabidopsis*, similar to that induced by the SA positive control, suggesting their role as SA elicitors. However, since the transgenic *Arabidopsis* carries only the *PR1* promoter, azomycin at all tested concentrations induced significantly lower GUS activity than that of JCK-8368. This suggests that JCK-8368 may contain additional compounds such as hydrolytic enzymes and auxin (**Supplementary Table S3**, **Supplementary Figure S10**) with stronger GUS-enhancing properties or that act additively or synergistically with azomycin to boost efficacy. Azomycin itself may also trigger defense-related genes beyond *PR1*.

The expression of PR genes serves as a key marker of plant immune activation (Jain and Khurana, 2018). The *PR1*, *PR2*, and *PR5* transcripts indicate SA signaling, while *PR3* is essential for the JA pathway (Thomma et al., 1998). These SA-dependent and JA-dependent pathways activate SAR and ISR, respectively (Zhang et al., 2020). Rather than directly targeting pathogens, these pathways enhance the innate defense of the plant, enabling quicker and stronger responses to future infections (Hammerschmidt, 2009; Kamle et al., 2020). SAR typically triggers localized infections and associates with hypersensitive responses, whereas ISR activates systemically, often through beneficial root-associated microbes (Hammerschmidt, 2009; Fu and Dong, 2013). In our study, CFA100 application significantly upregulates *PR1*, *PR3*, and *PR5* in apple seedlings, indicating that *Streptomyces* sp. JCK-8368 and its metabolite, azomycin, activate SAR and ISR. *PR3* (an ISR-related gene) was upregulated earlier (1.73-fold at 1 DAI), while *PR1* and *PR5* (SAR markers) peaked later but at higher levels (4.63-fold and 5.21-fold, respectively, at 2 DAI). This timing is notable, as the peak *PR1* and *PR5* expression coincided with the earliest observed disease suppression, suggesting that rapid activation of ISR may provide early pathogen inhibition, followed by a stronger SAR-mediated defense for sustained protection. *PR* proteins, especially those encoded by *PR1* and *PR5*, are well-established markers of salicylic acid–mediated resistance, often linked to reduced pathogen growth in various crops. While these findings imply a causal relationship between timely defense gene induction and disease suppression, further studies with pathogen population tracking or pathway-specific inhibitors are needed to confirm the mechanism. Changes in the expression levels of defense-related genes *PR1*, *PR2*, and *PR3* in A100-treated tomato seedlings reveal that azomycin can enhance the immune system of the plant, providing protection against pathogens. Azomycin treatments rapidly increased *PR1* and *PR2* expression (9.82-fold and 3.30-fold, respectively) at 1 DAI, while *PR3* peaked moderately (3.39-fold) at 2 DAI. These findings showed that azomycin mainly triggers the SAR pathway rather than ISR.

Our study showed that the effectiveness of azomycin depends on its application method: foliar spraying is more effective against the aerial pathogen *E. amylovora* TS3128, while soil drenching offers greater protection against the soilborne pathogen *R. solanacearum* SL341. This supports previous findings that matching the application method with the infection pathway of the pathogen enhances SAR activation and disease control (Conrath, 2006; Kim and Lim, 2023). Azomycin induced strong plant responses at low concentrations (in the nanogram range), suggesting its potential as a priming agent without phytotoxic effects. Other natural products also activate plant defenses at low concentrations, such as harpin proteins derived from *Erwinia* spp. and oxalic acid derived from *Aspergillus niger* (Dong et al., 1999; Yeon et al., 2023). Compared to high-dose applications, low-dose applications minimize costs and reduce potential negative effects on plant growth (Agathokleous et al., 2024; Teng et al., 2023; Velini et al., 2008). This study is the first to identify azomycin, a metabolite of *Streptomyces* sp. JCK-8368 is a dual-function agent that directly inhibits *E. amylovora* and *R. solanacearum* while priming plant immunity through the activation of SAR and ISR pathways. The dual functionality and low-dose efficacy of azomycin highlight its potential as a promising, sustainable, and eco-friendly biocontrol agent.

## 5 Conclusions

*Streptomyces* sp. JCK-8368 and its secondary metabolite azomycin demonstrated significant effectiveness in controlling tomato bacterial wilt and apple fire blight at low concentrations. Treatments with JCK-8368 (1,000-fold dilution) and azomycin (100 ng/ml) upregulated the expression of defense-related genes (*PR1*, *PR2*, *PR3*, and *PR5*), helping plants prepare for infection. At higher doses, azomycin caused temporary leaf yellowing in tomatoes, showing that the dose needs to be carefully adjusted. Azomycin worked best when applied in a way that matched the pathogen’s natural infection path. This is the first report showing that azomycin from *Streptomyces* spp. can trigger plant resistance to bacterial disease at nanogram-level concentrations, but comparisons with other resistance inducers are still needed. The study was limited to controlled conditions, so field trials are required to confirm results, check safety over time, and develop practical ways to apply these treatments in farming.

## Data Availability

The datasets presented in this study can be found in online repositories. The names of the repository/repositories and accession number(s) can be found in the article/**Supplementary Material**.
