# Supplementary material for: Biological control of tomato bacterial wilt and apple fire blight through the induced resistance of azomycin derived from Streptomyces sp. JCK-8368
Source: Front Plant Sci. 2025 Sep 26;16:1654826. doi: 10.3389/fpls.2025.1654826 (PMC12511057; doi:10.3389/fpls.2025.1654826)
Supplement: Supplementary file 1 [file DataSheet1.docx]

Supplementary Material

# Supplementary Figures and Tables

## Supplementary Tables

**Supplementary Table S1**. Primers for PCR and qRT-PCR

| **Gene** | **Primer** | **Sequence (5’-3’)** | **References** |
| --- | --- | --- | --- |
| *16S* rRNA | 27F | AGAGTTTGATCCTGGCTCAG | (Miller et al. 2013) |
|  | 1492R | GGTTACCTTGTTACGACTT |  |
| *PR1* | PR-1aF | GCTCAGCCGTAATACAATCCTCTC | (Shi et al. 2022) |
|  | PR-1aR | TACCCCCACTACTGCACCTCACT |  |
| *PR3* | MdPR3-1-QF | CTTTCGTTTGCTGCTGCTCGGTC |  |
|  | MdPR3-1-QR | TCTGGTGCACTTTCCCATCCTCC |  |
| *PR5* | PR5F | CAAGCAGCTTCCCTCCTCGGC |  |
|  | PR5R | GCCCCAGAAGCGACCAGACC |  |
| *EF-1α* | MdEF-1α-F | ATTCAAGTATGCCTGGGTGC |  |
|  | MdEF-1α-R | CAGTCAGCCTGTGATGTTCC |  |
| *PR1* | PR1F | GCCAAGCTATAACTACGCTACCAAC | (Song et al. 2010) |
|  | PR1R | GCAAGAAATGAACCACCATCC |  |
| *PR2* | PR2F | GGACACCCTTCCGCTACTCTT |  |
|  | PR2R | TGTTCCTGCCCCTCCTTTC |  |
| *PR3* | PR3R | AACTATGGGCCATGTGGAAGA |  |
|  | PR3R | GGCTTTGGGGATTGAGGAG |  |
| *Ubi* | UbiF | TCCATCTCGTGCTCCGTCT |  |
|  | UbiR | GAACCTTTCCAGTGTCATCAACC |  |

**Supplementary Table S2**. MICs of the fermentation filtrate obtained from *Streptomyces* sp. JCK-8368 and its antibacterial metabolite against phytopathogenic fungi

| **Phytopathogenic fungi** | **MIC** | |
| --- | --- | --- |
|  | **Fermentation filtrate (%)** | **Azomycin (µg/mL)** |
| *Botryosphaeria dothidea* | >10.00 ± 0.00 | 200.00 ± 0.00 |
| *Botrytis cinerea* | >10.00 ± 0.00 | 100.00 ± 0.00 |
| *Clarireedia homoeocarp* | 1.67 ± 0.64 | 6.25 ± 0.00 |
| *Rhizoctonia solani* AG 2-2 | 0.83 ± 0.32 | 33.33 ± 12.73 |

Data are expressed as mean ± standard deviation of three replicates.

MICs, Minimum inhibitory concentrations

**Supplementary Table S3**. Enzyme activity of strain JCK-8368

| **Enzyme assayed for** | **JCK-8368** |
| --- | --- |
| Control | - |
| Alkaline phosphatase | + |
| Esterase (C4) | + |
| Esterase Lipase (C8) | + |
| Lipase (C14) | - |
| Leucine arylamidase | + |
| Valinearyl lamidase | + |
| Cysteine arylamidase | + |
| Trypsin | + |
| α-Chymotrypsin | + |
| Acid phosphatase | + |
| Naphthol-AS-BI-phosphohydrolase | + |
| α-Galactosidase | - |
| β-Galactosidase | - |
| β-Glucuronidase | - |
| α-Glucosidase | - |
| β-Glucosidase | - |
| N-acetyl-β-gucosaminidase | + |
| α-Mannosidase | + |
| α-Fucosidase | - |
| Protease | - |
| Amylase | - |
| Gelatinase | - |
| Cellulase | - |

+ Positive; - negative

## Supplementary Figures

Supplementary Figure S1. Scheme isolation of azomyci
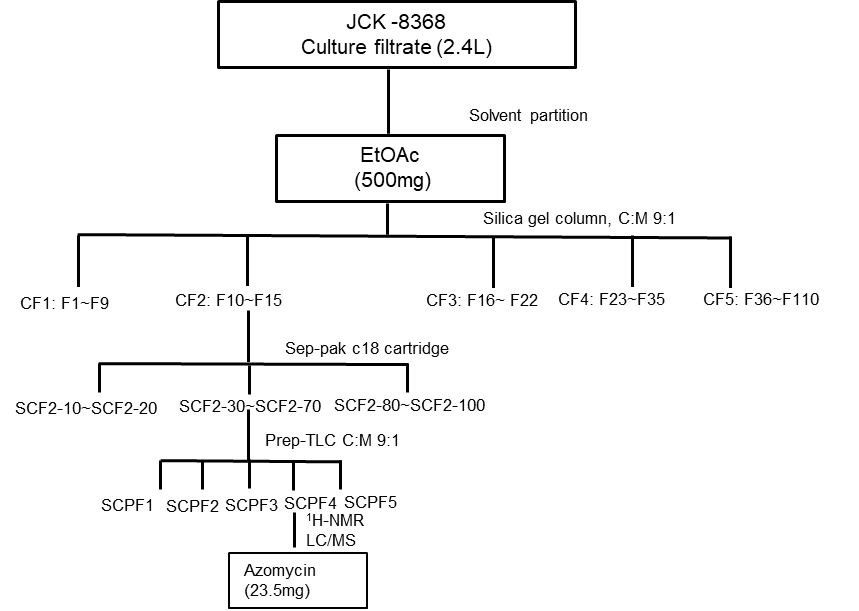
n from the ethyl acetate extract of JCK-8368

The JCK-8368 culture broth was filtered through sterile gauze to collect the cell-free (CF) supernatant. This CF supernatant was partitioned with organic solvents including ethyl acetate (EtOAc) and butanol (BuOH). The EtOAc, BuOH, and aqueous layers were individually concentrated to dryness and re-dissolved to 50 mg/mL in acetone, methanol, and distilled water (DW), respectively. They were employed in thin-layer chromatography (TLC, Kiesel gel 60 GF254, 0.2 mm film thickness, E. Merck, Darmstadt, Germany) for bioautography against *E. amylovora* TS3128. TLC plates were developed using CHCl3: MeOH (9:1, v/v). Four repetitions were done with markings under UV light at 254 nm and 360 nm. One TLC plate served as a reference and was analyzed with *p*-anisaldehyde. Remaining TLC plates were dried and sterilized under UV light for 15 minutes on each side. These plates were then overlaid with TSA containing 0.5% *E. amylovora* TS 3128 (3.3×10^5^ CFU/mL) on Petri dishes and incubated at 30°C for 24 hours. Inhibition zones appeared as clear spots against a milk-white backdrop, with their Rf values compared to the reference TLC plate. The EtOAc layer with the highest activity was selected for further experiments. The EtOAc extract (0.5 g) of *Streptomyces* sp. JCK-8368 was subjected to chromatography on a silica gel column (2.5 × 80 cm) packed with 50 g of silica gel (Kiesel gel 60, 230–400 mesh: E. Merck, Darmstadt, Germany) using CHCl_3_: MeOH (9:1, v/v) as the eluent. The fractions were visualized by TLC (Kiesel gel 60 GF_254_, 0.2 mm film thickness, E. Merck, Darmstadt, Germany) with CHCl_3_: MeOH (9:1, v/v) as mobile phase. The chromatography yielded 110 fractions (F1-F110). Based on the TLC profiles, the fractions were combined into five groups, namely CF1(F1-F9), CF2(F10-F15), CF3(F16-F22), CF4(F23-F35) and CF5(F36-F110). Each group was dissolved in 1 mL of acetone and tested for antibacterial activity against *E. amylovora* TS 3128, with 1% acetone used as the untreated control.

Group CF2 (148.35 mg) showed antibacterial activity and were passed through a Sep-Pak C_18_ cartridge (Sep-Pak Vac 35 cc, 10 g; Waters Corporation, Milford, MA, USA) and eluted with MeOH:water (10:90, 20:80, 30:70, 40:60, 50:50, 60:40, 70:30, 80:20, 90:10, 100:0, v/v; 100 mL of each). Preparative thin-layer chromatography (Prep-TLC) was performed on Kieselgel 60 GF254 (0.5 mm film thickness; E. Merck, Darmstadt, Germany) using CHCl_3_:MeOH (9:1, v/v) as an eluent. Moreover, TLC (Kieselgel 60 GF254, 0.2 mm film thickness; E. Merck, Darmstadt, Germany) was performed to visualize the fractions, with CHCl_3_:MeOH (9:1, v/v) serving as the mobile phase. Fractions with similar retardation factor (Rf) values were combined according to TLC profiles. Based on the TLC profiles, the fractions were combined into three groups, namely G1(SCF2-10~SCF2-20), G2(SCF2-30~SCF2-70), and G3(SCF2-80~SCF2-100). After dissolving each group in acetone, the antibacterial activity against *E. amylovora* TS3128 was assessed; 1% acetone served as the untreated control. Group G2 (97.45 mg) exhibited antibacterial activity and was further purified by preparative thin layer chromatography (TLC) on Kiesel gel 60 GF_254_ (0.5 mm film thickness, E. Merck, Darmstadt, Germany) with CHCl_3_: MeOH (9:1, v/v). The silica gel was scraped off from the TLC plate, and fraction (SCPF4, 23.50 mg) was obtained. This fraction was eluted with acetone, redissolved in 100 µL of acetone, subjected to bioassay, and showed activity.

**C_3_H_2_N_3_O_2_^-^**

Supplementary Figure S2. UHPLC-Q-Orbitrap MS spectra of SCPF4 (C_3_H_2_N_3_O_2_^-^, negative mode)


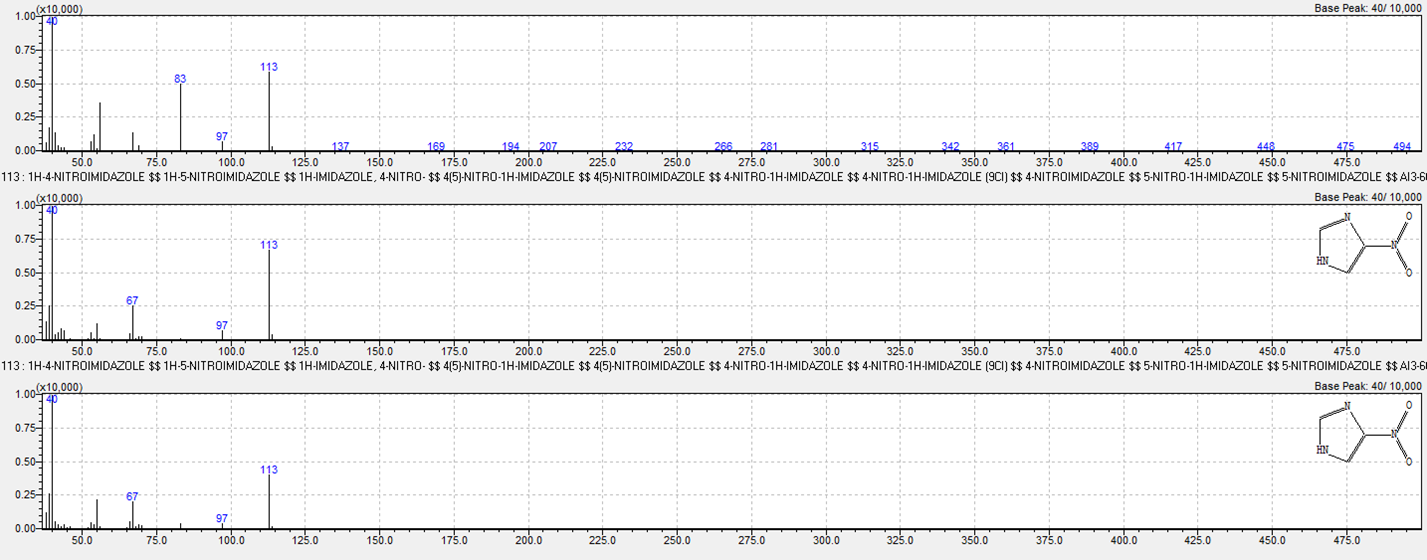


4-nitroimidazole

SCPF4

Supplementary Figure S3. GC-MS spectra of SCPF4 and 4-nitroimidazole


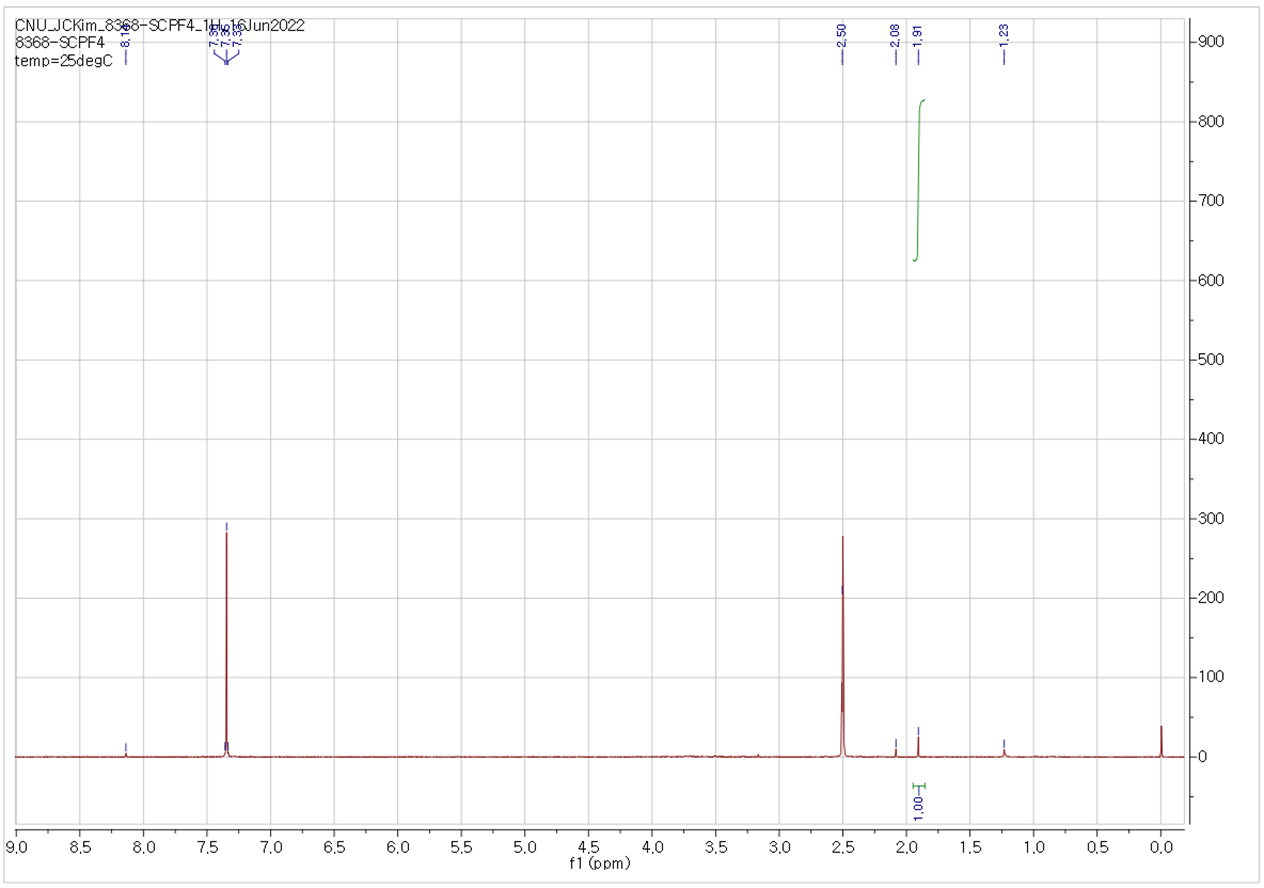


Supplementary Figure S4. ^1^H-NMR spectra of SCPF4

| **Azomycin** | |
| --- | --- |
| **Peak area (µV*sec)** | **Concentration(µg/mL)** |
| 3072886±198 | 94.15±0.08 |

 Supplementary Figure S5. Quantitative concentration of azomycin producing from *Streptomyces* sp. JCK-8368 by HPLC. (A) Calibration curve of azomycin (B) Peak area and concentration of azomycin in culture filtrate of JCK-8368

**B**

**A**


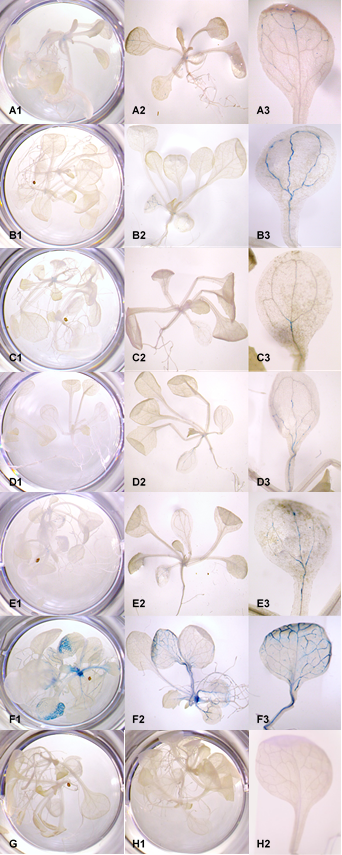


Supplementary Figure S6. GUS activity of azomycin in transgenic *Arabidopsis thaliana* at different concentrations including (A) 1000 ng/mL, (B) 100 ng/mL, (C) 20 ng/mL, (D) 10 ng/mL, (E) 1 ng/mL, (F) Salicylic acid 0.1 mM, (G) SDW, (H) Acetone 1


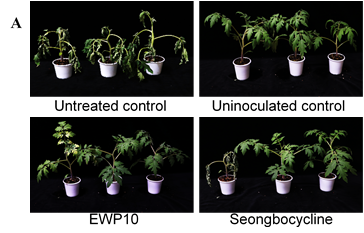


**Supplementary** **Figure S7**. Disease control efficacy of EWP10, a wettable powder formulation containing ethyl acetate extract of JCK-8368, against tomato bacterial wilt caused by *R. solanacerum* SL341; Seong, Seongbocycline. Error bars indicate standard errors. *(*p* < 0.05) represents significant differences by the Tukey’s HSD test.

**
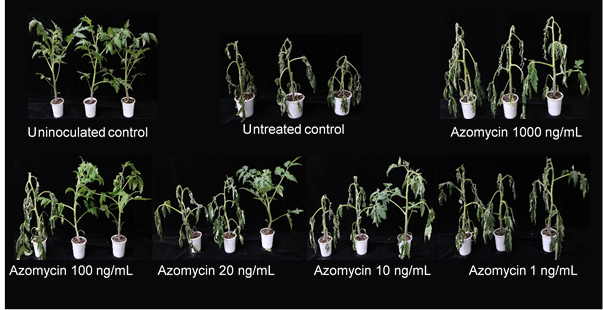
**

**Supplementary** **Figure S8**. Pretreatment of azomycin against tomato bacterial wilt caused by *R. solanacerum* SL341


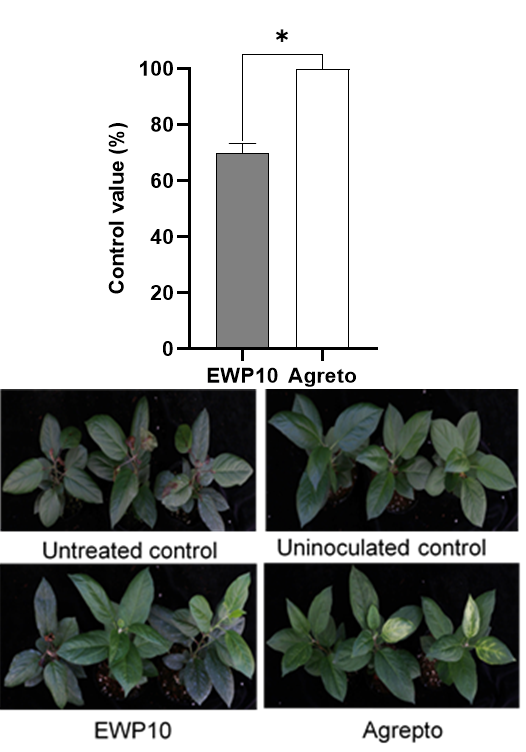


**Supplementary** **Figure S9**. Disease control efficacy of EWP10, a wettable powder formulation containing ethyl acetate extract of JCK-8368, against apple fire blight caused by *E. amylovora* TS3128, formulation containing the ethyl acetate partition of JCK-8368 fermentation broth. Error bars indicate standard errors. *(*p* < 0.05) represents significant differences by the Tukey’s HSD test.

B

A

**Supplementary** **Figure S10**. (A) Standard curve of IAA, (B) IAA production of JCK-8368

**References**

Miller, C.S., Handley, K.M., Wrighton, K.C., Frischkorn, K.R., Thomas, B.C., Banfield, J.F. (2013). Short-read assembly of full-length 16S amplicons reveals bacterial diversity in subsurface sediments. *PLoS One* 8(2), e56018.

Shi, J., Jiang, Q., Zhang, S., Dai, X., Wang, F., Ma, Y. (2022). MIR390 is involved in regulating anthracnose resistance in apple. *Plants* 11(23), 3299.

Shirling, E.T., and Gottlieb, D. (1966). Methods for characterization of *Streptomyces* species. *Int J Syst Bacteriol* 16(3), 313-340 doi: <https://doi.org/10.1099/00207713-16-3-313>.

Song, Y.Y., Zeng, R.S., Xu, J.F., Li, J., Shen, X. and Yihdego, W.G. (2010). Interplant communication of tomato plants through underground common mycorrhizal networks. *PloS one*, 5(10), p.e13324.
